# Rapid Interpretation of Protein Backbone Rotation Dynamics Directly from Spin Relaxation Data

**DOI:** 10.1021/acs.jpclett.4c01800

**Published:** 2024-10-01

**Authors:** Ricky Nencini, Efstathia Mantzari, Amanda E. Sandelin, O. H. Samuli Ollila

**Affiliations:** † Institute of Biotechnology, University of Helsinki, Helsinki 00014, Finland; ‡ Division of Pharmaceutical Biosciences, Faculty of Pharmacy, University of Helsinki, Helsinki 00014, Finland; ¶ VTT Technical Research Centre of Finland, Espoo 02044, Finland; § Division of Pharmacology and Pharmacotherapy, Faculty of Pharmacy, University of Helsinki, Helsinki 00014, Finland

## Abstract

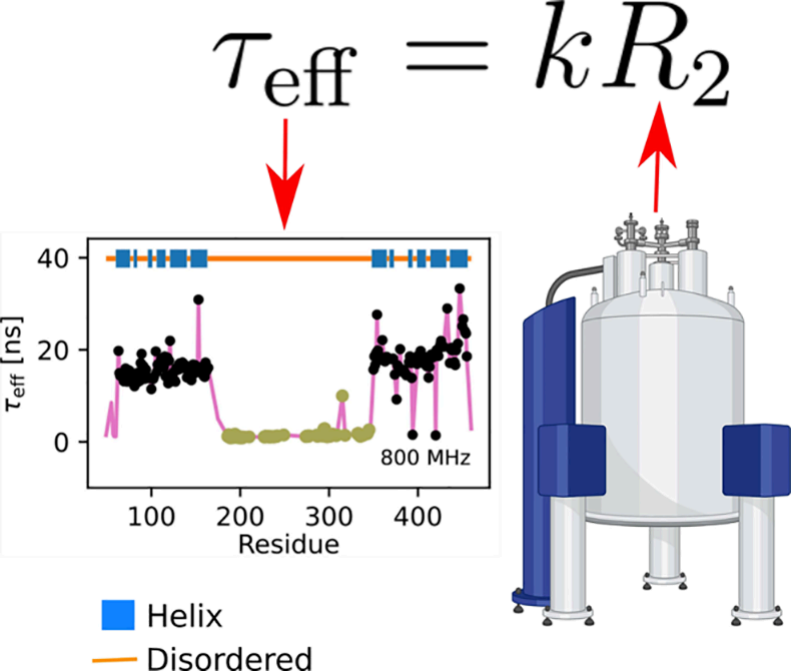

Besides their structure, dynamics is pivotal for protein functions, particularly for intrinsically disordered proteins (IDPs) that do not fold into a fixed 3D structure. However, the detection of protein dynamics is difficult for IDPs and other disordered biomolecules. NMR spin relaxation rates are sensitive to the rapid rotations of chemical bonds, but their interpretation is arduous for IDPs or molecular assemblies with a complex dynamic landscape. Here we demonstrate numerically that the dynamics of a wide range of proteins, from short peptides to partially disordered proteins and peptides in micelles, can be characterized by calculating the total effective correlation times of protein backbone N–H bond rotations, τ_eff_, from experimentally measured transverse ^15^N spin relaxation rates, *R*_2_, using a linear relation. Our results enable the determination of magnetic-field-independent and intuitively understandable parameters describing protein dynamics at different regions of the sequence directly from experiments. A practical advance of the approach is demonstrated by analyzing partially disordered proteins in which rotations of disordered regions occur with timescales of 1–2 ns, independent of their size, suggesting that rotations of disordered and folded regions are uncoupled in these proteins.

High-resolution three-dimensional (3D) structures of proteins, widely used in drug design and other biotechnical applications, can now be also solved computationally,^1^ but it is still difficult to resolve their dynamics. This is particularly relevant for disordered residues, comprising 20–60% of proteins,^2^ which do not fold to fixed 3D structures but exhibit large conformational fluctuations, forming intrinsically disordered proteins (IDPs). Fully and partially disordered proteins bear heterogeneous dynamics that are complicated to characterize experimentally and theoretically.^3,4^ Furthermore, peptides associated with lipid or surfactant aggregates form another important protein class, the dynamics of which is difficult to characterize.^5^ Better methods to characterize the dynamics of such systems are expected to benefit many fields where IDPs and peptide aggregates with lipids or other biomolecules are relevant, ranging from the design of drugs targeting disordered proteins^6,7^ and novel materials based on silk proteins^8^ to disordered peptides with potential antimicrobial activity^9^ and nanodiscs with biomedical relevance.^10^ Here we present a rapid way of determining average rotational timescales of residues in folded and disordered proteins, as well as in peptides in micelles, using data that can be routinely measured experimentally and is widely available in the literature.

Rotational dynamics of individual residues in proteins can be probed using nuclear magnetic resonance (NMR) experiments that detect spin relaxation rates, *R*_1_ and *R*_2_, and the heteronuclear Overhauser effect (hetNOE) of backbone ^15^N atoms. For folded proteins, residual dynamics can be described by order parameters and timescales representing internal and overall motions, which can then be solved from experimental spin relaxation rates using the so-called Lipari–Szabo approach.^11^ However, the dynamics of proteins with disordered regions or peptides in complex biomolecular environments cannot be described by just a few parameters; thus, interpretation of their dynamics requires molecular dynamics (MD) simulations or other elaborate models.^3−5^

Interpretation of protein or peptide rotational dynamics from NMR experiments is usually based on Redfield equations that relate the spin relaxation rates to molecular dynamics,^12,13^
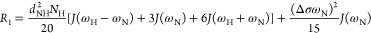
1
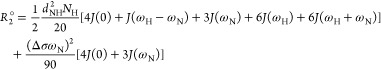
2
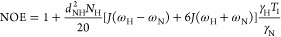
3where ω_H_ and ω_N_ are the Larmor frequencies of ^1^H and ^15^N, respectively, *N*_H_ = 1 is the number of protons in the N–H bond, and Δσ = −160 ppm is the chemical shift anisotropy.^14^ The dipolar coupling constant is defined as *d*_NH_ = , where μ_0_ is the vacuum permeability, ℏ is the reduced Planck constant, γ_H_ and γ_N_ are the gyromagnetic constants of ^1^H and ^15^N, respectively, and the average cubic length is calculated as ⟨*r*_NH_^3^⟩ = 0.101 nm. Spectral density, *J*(ω), is the Fourier transform of the second-order rotational correlation function of the N–H bond vector, *g*(*t*). Assuming multiexponential decay for *g*(*t*) with *N* number of timescales τ_*i*_ with the weights of α_*i*_, the spectral density can be written as
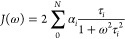
4Timescales, τ_*i*_, and their weights, α_*i*_, are typically solved by fitting eqs 1–4 to the experimental data^15,16^ or using MD simulations.^3−5,17^ Notably, the transverse spin relaxation time in eq 2 is given without a chemical exchange contribution, *R*_2_^°^ = *R*_2_ – *R*_ex_, where *R*_ex_ arises from chemical or conformational kinetic processes, where nuclear spins transfer between different chemical environments on microsecond to millisecond timescales that affect experimental *R*_2_ values but are not captured by eq 2.

Here we describe the rotational dynamics of a bond using the total effective correlation time, τ_eff_ = ∑_0_^*N*^α_*i*_τ_*i*_, which is the weighted average of all timescales in a multiexponential decay assumed for *g*(*t*) and equals the area between the correlation function and *x*-axis. Notably, τ_eff_ is the total effective correlation time containing all rotational timescales, including internal and overall rotations. This definition has been used previously, for example, by Levy et al.,^18^ but differs slightly from the effective correlation times of molecular internal motions used in the Lipari–Szabo approach^11,13^ and for lipids in bilayers,^19^ where effective correlation times include only internal motions with respect to molecular axes. Interestingly, by substituting eq 4 into eq 2 and using the relation *J*(0) = 2τ_eff_, the Redfield equation for *R*_2_^°^ can be written as
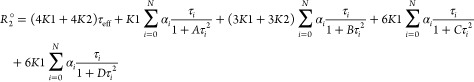
5where *K*1 = , +*K*2 = , *A* = (ω_H_ – ω_N_)^2^, *B* = (ω_N_)^2^, *C* = (ω_H_)^2^, and *D* = (ω_H_ + ω_N_)^2^. The first term of eq 5 gives a linear relation between *R*_2_^°^ and τ_eff_, but the magnitudes of other terms are difficult to evaluate in systems with unknown distributions of rotational timescales, represented as τ_*i*_ values and their weights α_*i*_ in eq 4.

Inspired by eq 5, we numerically investigated the relation between *R*_2_^°^ values and the total effective correlation times in MD simulations. Figure 1 shows τ_eff_ values and spin relaxation rates, *R*_1_, *R*_2_^°^, and hetNOE, calculated separately for each residue from MD simulations of a wide range of different protein systems, including globular and multidomain proteins, as well as peptide–micelle complexes.^3−5,17^ Indeed, we observe a practically linear relation between τ_eff_ and *R*_2_^°^, suggesting that only the first term in eq 5 is significant for all different types of proteins and peptides with different dynamic landscapes.^3−5,17^ The magnitudes of the different terms in eq 5, shown in Figure S1, reveal that the linear term indeed dominates and nonlinear terms have significant contribution only on timescales below approximately 3 ns. On the other hand, the relations between τ_eff_ and *R*_1_ or hetNOE values are more complicated; Figure 1 reveals ambiguous relations between these parameters and N–H bond rotational dynamics. In addition, Figure 1 shows numerically solved lines from eqs 1–3, assuming that the N–H bond rotation is dictated by only one timescale, which then equals the total effective correlation time. This approach, denoted here as the 1-timescale approximation, also gives results close to simulations for *R*_2_^°^, while significant deviations are observed for *R*_1_ and hetNOE.

**Figure 1:**
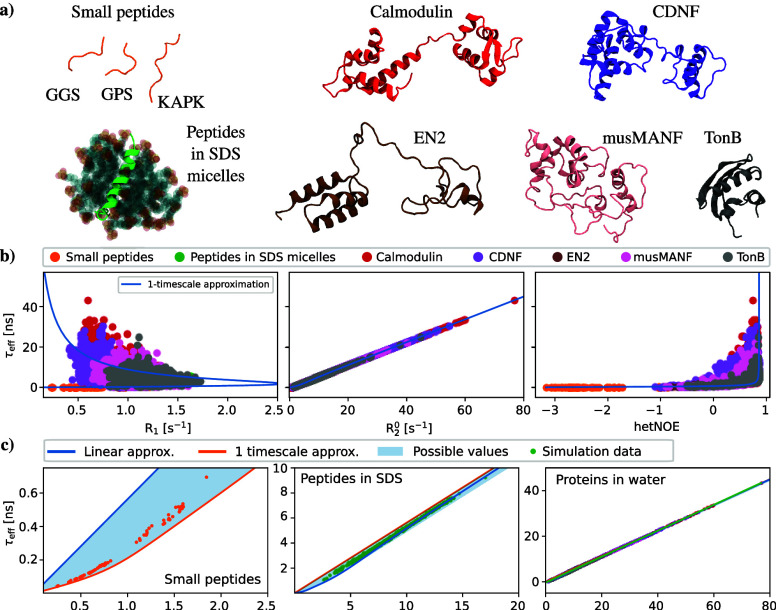
(a) Representative conformations of proteins and peptides in MD simulations. (b) Effective correlation times, τ_eff_, as functions of spin relaxation rates from MD simulations of different types of systems (dots) and 1-timescale approximation substituted into Redfield equations (blue solid lines). (c) Effective correlation times, τ_eff_, as a function of *R*_2_^0^ shown separately for different types of systems and different *x*-axis scales.

The results in Figure 1 suggest that effective correlation times can be directly derived from *R*_2_^°^ values using the first term of eq 5, denoted here as the linear approximation. However, a more detailed comparison in Figure 1b shows that the linear approximation slightly overestimates effective correlation times that are smaller than approximately 3 ns, in line with Figure S1, showing magnitudes of different terms in eq 5. On the other hand, the 1-timescale approximation gives slightly better results for the fast timescales. The blue-shaded regions in Figure 1b show the numerically solved possible range of effective time values that can appear for the given *R*_2_^°^. In conclusion, linear approximation sets the upper limit for possible τ_eff_ values corresponding to a given *R*_2_^°^ value, while the 1-timescale approximation sets the lower limit for τ_eff_ values of residues with rapid rotations and asymptotically approaches the linear approximation for longer timescales.

Accuracies of linear and 1-timescale approximations to determine τ_eff_ values directly from *R*_2_^°^ values are quantified against simulation data in Figures S2 and S3. Linear approximation systematically overestimates τ_eff_ values, particularly for low fields (up to 1.5 ns for 400 MHz, less than 0.5 ns for fields above 600 MHz) and residues with fast timescales below approximately 3 ns (Figure 1b). The 1-timescale approximation has a slight tendency to underestimate τ_eff_ values, but it can be considered to be slightly more accurate than the linear approximation, particularly for residues with rapid rotations (Figure 1b and Figures S2 and S3).

Although the accuracy of linear approximation is slightly less than that of the 1-timescale approximation, it enables very rapid interpretation of total effective correlation times directly from experimental *R*_2_^°^ values using the equation
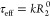
6where *k* = (4*K*1 + 4*K*2)^−1^. To simplify this in practice, we have listed numerical *k* values with the most commonly used magnetic fields in Table 1. In addition, we provide numerical results where overestimation of τ_eff_ values in linear approximation are corrected by fitting the equation τ_eff_ = *mR*_2_^°^ + *c* to the MD simulation data, which improves the accuracy also with respect to the 1-timescale approximation (Figure S4). For the 1-timescale approximation, we provide a simple Python function^20^ that takes *R*_2_ values and the magnetic field as input and returns the effective rotational time by numerically solving the inverse function of eq 5.

**Table 1:** Proportionality Constants, *k* = (4*K*1 + 4*K*2)^−1^, of Linear Approximation and Parameters (*m* [×10^–9^ s^2^] and *c* [ns]) Extracted by Fitting to MD Simulation Data at Different Magnetic Fields^a^

ω_H_ [MHz]	*k* [×10^–9^ s^2^]	τ_eff_ [ns] = *mR*_2_^°^ + *c*
300	0.84	τ_eff_ = 0.85*R*_2_^°^ – 1.52
360	0.81	τ_eff_ = 0.83*R*_2_^°^ – 1.26
400	0.80	τ_eff_ = 0.81*R*_2_^°^ – 1.11
500	0.75	τ_eff_ = 0.76*R*_2_^°^ – 0.83
600	0.69	τ_eff_ = 0.71*R*_2_^°^ – 0.64
700	0.64	τ_eff_ = 0.65*R*_2_^°^ – 0.51
720	0.63	τ_eff_ = 0.64*R*_2_^°^ – 0.49
750	0.61	τ_eff_ = 0.62*R*_2_^°^ – 0.46
800	0.59	τ_eff_ = 0.60*R*_2_^°^ – 0.41
850	0.56	τ_eff_ = 0.57*R*_2_^°^ – 0.38
900	0.54	τ_eff_ = 0.54*R*_2_^°^ – 0.34
950	0.51	τ_eff_ = 0.52*R*_2_^°^ – 0.31
1000	0.49	τ_eff_ = 0.50*R*_2_^°^ – 0.29
1200	0.41	τ_eff_ = 0.41*R*_2_^°^ – 0.22
1500	0.31	τ_eff_ = 0.32*R*_2_^°^ – 0.16

a Magnetic field strengths are expressed in terms of proton Larmor frequency, ω_H_.

To demonstrate the advantage of rapid analysis of molecular dynamics directly from experimental spin relaxation data, we used the 1-timescale approximation to calculate total effective correlation times from experimental *R*_2_ values available in the literature^3,21−26^ for partially disordered proteins containing 114–411 residues, as shown in Figure 2. These proteins are beyond the scope of standard approaches used to interpret spin relaxation experiments because partially disordered proteins bear a complex dynamic landscape that needs to be described by a large number of parameters.^3^ MD simulations could be used, but they require a careful selection of simulation parameters and a substantial amount of computational resources.^3,4^Figure 2 shows that the total effective correlation times of folded regions vary between approximately 5 and 17 ns with a slight dependence on the size of the region (Pearson correlation coefficient 0.66, *p*-value 0.05). On the other hand, effective correlation times of disordered regions are approximately 1–2 ns, independent of the size of the protein or the disordered part (Pearson correlation coefficient 0.17, *p*-value 0.66). This suggests that the dynamics of folded and disordered parts are uncoupled in these proteins, similarly to the TonB protein that was previously analyzed by combining NMR experiments and MD simulations.^3,27^

**Figure 2:**
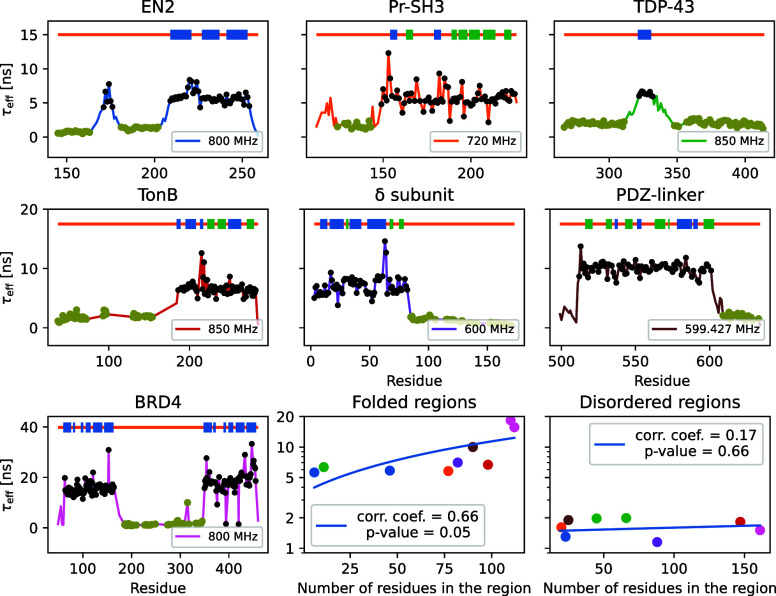
Effective correlation times of partially disordered proteins calculated from experimental *R*_2_ values from the literature (EN2^21^ – blue, Pf-SH3^22^ – orange, TDP-43^23^ – green, TonB^3^ – red, δ-subunit^24^ – violet, PDZ-linker^25^ – brown, and BRD4^26^ – pink). Secondary structures of each protein are illustrated at the top of each panel with color coding (blue: α helix, green: β sheet; orange: disordered). All points are connected by colored lines. Residues that are manually assigned to belong to disordered regions are highlighted with green points, and residues assigned to folded regions are indicated with black points. Average correlation times for these folded and disordered regions as a function of their size, together with the Pearson correlation analysis, are shown in the bottom right corner. Color coding for proteins is the same as that in other plots.

For folded proteins, results from the linear approximation presented in this work are in line with the standard Lipari–Szabo analysis^28^ in Figure S5. In conclusion, our approach enables rapid determination of effective correlation times of N–H bonds, τ_eff_, from experimental *R*_2_ values for a wide range of proteins. Effective correlation times describe protein dynamics in an intuitively understandable way also for scientists without NMR expertise. Furthermore, their value does not depend on the magnetic field, in contrast to *R*_2_, thereby enabling straightforward comparison between data measured at different magnetic fields and proteins. This is particularly useful for IDPs and proteins complexed with lipids that are beyond the scope of standard methods to interpret spin relaxation times.

To further utilize the possibility to rapidly extract magnetic -field-independent descriptors of molecular dynamics from experimental spin relaxation data, we use the linear approximation to calculate total effective correlation times for all proteins from the BMRB database (https://bmrb.io/)^29^ that have experimental *R*_2_ values available in four different magnetic fields; see Figure 3. Effective correlation times calculated from different magnetic fields are generally in good agreement with each other, particularly with higher fields and lower effective correlation time values. This can be explained by the increased experimental resolution in higher fields and the higher sensitivity of *R*_2_ values to small errors in *T*_2_ (inverse of *R*_2_) with slow dynamics (i.e., small *T*_2_ leading to large *R*_2_). However, inconsistencies between magnetic fields are observed in some cases. For example, *C. symbiosum* archaeal parvulin, CsPinA (BMRB 18864), exhibits inconsistencies for some residues between 400 and 600 MHz, 400 and 700 MHz, 600 and 700 MHz, and 500 and 700 MHz. These may arise from inaccuracies in spin relaxation measurements or the influence of chemical exchange, *R*_ex_, on *R*_2_ values.^37^

**Figure 3:**
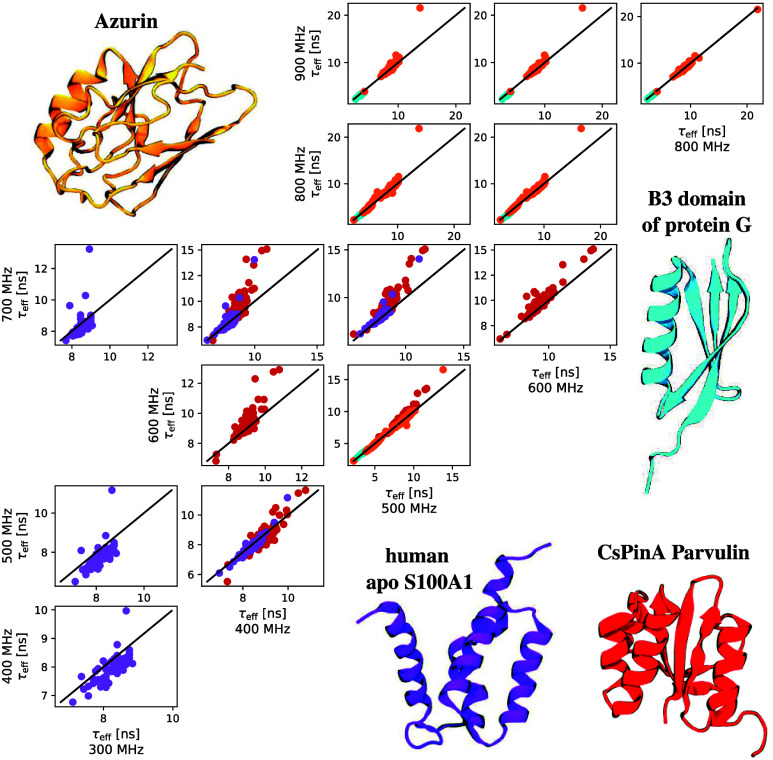
Effective correlation times calculated using linear approximation for all proteins with *R*_2_ values available in four different magnetic fields in the BMRB database:^29^ azurin (BMRB: 6243,^30^ PDB: 1AZU(31)), B3 domain of protein G (BMRB: 26845,^32^ PDB: 1IGD(33)), human apo S100A1 (BMRB: 16360, PDB: 2L0P(34)), and CsPinA parvulin (BMRB: 18864,^35^ PDB: 2M1I). Protein structures are taken from the Protein Data Bank (PDB).^36^ Data points and structures are labeled with the same color for each protein. The black lines show equal values with both magnetic fields.

To investigate the influence of chemical exchange on the magnetic field dependence of effective correlation time, we calculated τ_eff_ values with the chemical exchange correction using eq S17 in the Supporting Information and available experimental *R*_2_ values at different fields for CsPinA^35^ (Figure 4) and the B3 domain of protein G^32^ (Figure S6). Results for CsPinA without the correction term show systematic magnetic field dependence of τ_eff_ values, particularly around residue 50 in Figure 4a. Magnetic field dependence disappears after applying the correction term (Figure 4 b), and residues around residue 50 with significant chemical exchange contributions agree with those obtained in the literature using Lipari–Szabo analysis with data recorded at 500 and 700 MHz^35^ (Figure 4c). This indicates that the magnetic field dependence of τ_eff_ for CsPinA can be explained by the chemical exchange term. On the other hand, for the B3 domain of protein G, we observed systematically slightly lower τ_eff_ values with 800 and 900 MHz than with 500 and 600 MHz that cannot be explained by the chemical exchange correction (Figure S6). In conclusion, the magnetic field dependence of τ_eff_ can be explained by the chemical exchange in some cases and then used to determine the size of the chemical exchange contribution to *R*_2_ values. However, such interpretations should be done with caution, because magnetic field dependence may arise also from inaccuracies in the experimental data, as indicated in another study.^37^

**Figure 4:**
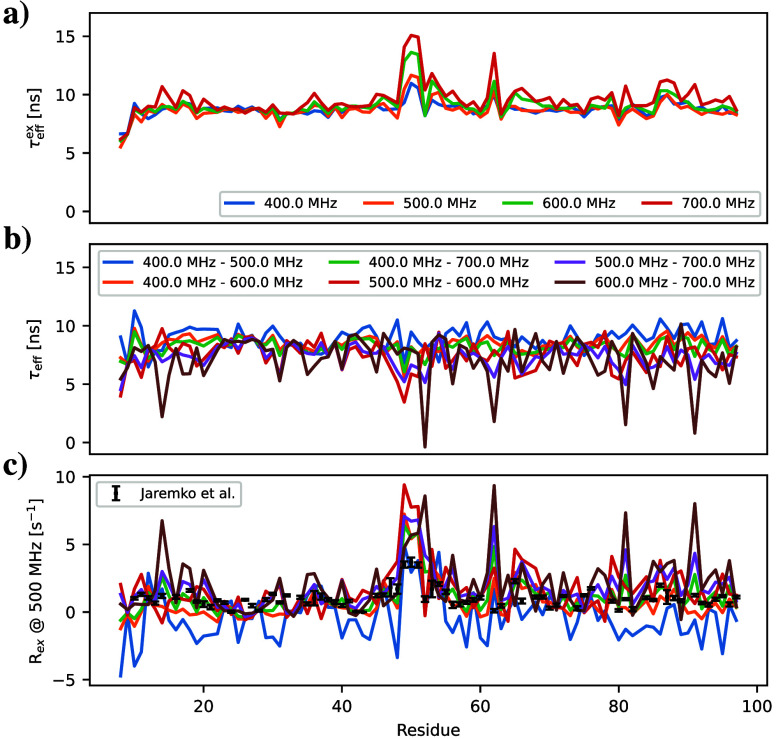
Effective correlation times for CsPinA determined from *R*_2_ at different fields (a) without and (b) with the correction term for chemical exchange. (c) The magnitude of the chemical exchange term at 500 MHz from our analysis and literature.^35^

In this Letter, we provide a numerically solved linear relation between the total effective rotational correlation time of protein backbone N–H bonds, τ_eff_, and the experimentally detectable transverse spin relaxation rate *R*_2_. The effective correlation time is a magnetic-field-independent and intuitively understandable parameter describing protein dynamics. This enables rapid interpretation of protein and peptide rotational dynamics directly from *R*_2_ values measured in one magnetic field without further modeling. Similar analyses might be possible by determining *J*(0) values from spectral density mapping,^38,39^ but this would require 3–6 experimental parameters per residue, while our approach requires only *R*_2_ values. Our results are valid for a wide range of systems that are beyond the scope of standard methods used to interpret molecular dynamics from spin relaxation experiments, from short peptides in water and micelles to large multidomain proteins with disordered regions. However, caution should be exercised when the chemical exchange contribution to experimental *R*_2_ values is unknown, because it may affect the results. Nevertheless, residues affected by the chemical exchange gave longer τ_eff_ values when the specific correction term was not used (e.g., residues around residue 50 in Figure 4a), indicating slower dynamics for these residues, which is a qualitatively correct interpretation for the effect of chemical exchange on rotational dynamics.

We present the linear relation between backbone N–H bond effective rotational correlation times and *R*_2_ values as a new route to characterize protein and peptide dynamics experimentally. This is a particularly promising approach for multidomain proteins, IDPs, and protein or peptides complexed with lipids or other biomolecules whose dynamics cannot be interpreted using standard approaches. We anticipate potential practical applications in a wide range of fields in which such systems play significant roles. The presented approach can be used to investigate, for example, how drug binding affects dynamics of IDPs,^6^ rotation of peptides in lipid nanodiscs with potential pharmaceutical applications,^10^ or dynamics of silk proteins forming novel materials.^8^ Furthermore, we provide a tool to immediately interpret the average rotational dynamics of residues of any kind of protein system from a spin relaxation experiment with only one magnetic field, contributing a significant practical advance in the interpretation of NMR experiments over a wide range of fields. This ability to transfer spin relaxation rates to intuitively understandable parameters describing protein dynamics makes NMR results accessible for a wider audience.

## Supporting Information Available

Additional simulation and theoretical details; additional results and figures on different contributions to *R*_2_ values, the accuracy of the results, comparison with the Lipari–Szabo approach, the influence of chemical exchange contribution, and magnetic field dependence (PDF)
Transparent Peer Review report available (PDF)
