# Supplementary material for: Rapid Interpretation of Protein Backbone Rotation Dynamics Directly from Spin Relaxation Data
Source: J Phys Chem Lett. 2024 Oct 1;15(40):10204–9. doi: 10.1021/acs.jpclett.4c01800 (PMC11480883; doi:10.1021/acs.jpclett.4c01800)
Supplement: Supplementary file 1 — jz4c01800_si_001.pdf [file jz4c01800_si_001.pdf]

Supplementary information: Rapid interpretation of protein backbone rotation dynamics directly from spin relaxation data

Ricky Nencini,[†,‡] Efstathia Mantzari,[¶,†] Amanda Sandelin,[§,†] and O. H. Samuli Ollila*,[¶,†]

†*Institute of Biotechnology, University of Helsinki, Helsinki, 00014, Finland*

‡*Division of Pharmaceutical Biosciences, Faculty of Pharmacy, University of Helsinki, Helsinki, 00014, Finland*

¶*VTT Technical Research Centre of Finland, Espoo, 02044, Finland*

§*Division of Pharmacology and Pharmacotherapy, Faculty of Pharmacy, University of Helsinki, Helsinki, 00014, Finland*

E-mail: samuli.ollila@helsinki.fi

Supplementary methods

Simulation details

Previously published MD simulations for peptides in micelles[1] and proteins (calmodulin, CDNF, EN2, MANF, and TonB)[2] were used. Small peptides with the sequences of $(GGS)_3$, $(GPS)_3$ and $K(AP)_5K$ were simulated using GROMACS version 2022.2[3] and DESAMBER[4] force field with TIP4P-D water model.[5] Initial configuration for each peptide was generated using ProBuilder software (`https://www.ddl.unimi.it/vegaol/probuilder.htm`), solvated to a cubic box with 12000, 16396, 28364 water molecules for $(GGS)_3$, $(GPS)_3$ and $K(AP)_5K$, respectively. Ions were added to mimic the 40 mM sodium chloride concentration and to neutralize the total charge of systems whenever needed. Systems were simulated for 1 μs with the timestep of 2 fs. Temperature was coupled using velocity rescaling with a stochastic term at 298 K (v-rescale) and pressure coupling performed with isotropic Parrinello-Rahman barostat at 1 bar. PME was used to calculate electrostatic interactions at distances longer than 1.0 nm and Lennard-Jones interactions were cut off at 1.2 nm. Five replicas were simulated for each peptide from different initial structures, taken from five timepoints (every 200ns) of the initial 1 μs MD trajectory. Simulation files and calculated correlation functions for short peptides are available at Ref. 6.

Spin relaxation times were calculated as described previously.[1,7] Shortly, second order rotational correlation functions of N-H bonds were calculated from trajectories using rotacf function in Gromacs.[8] These functions were then fitted to a sum of 100 exponentially decaying functions with predefined timescales, τ_i, equidistantly spaced in logarithmic scale between 1 fs and 100 ns.

$$C_{\text{fit}}(t) = \sum_{i=1}^{N} \alpha_i e^{-t/\tau_i}, \tag{S1}$$

where α_i is the weight with which the i-th exponential decay is present in the correlation

function. Spectral densities were then calculated using Eq. 4 and substituted to Eqs. 1-3.

Solving 1-timescale approximation

To numerically solve the relation between τ_{eff} and R_2 with 1-timescale approximation, we substitute only one timescale (τ with $\alpha = 1$) into Eq. 5, rewrite the equation into a polynomial form, and use numpy package poly1d to find roots of this 9th-order polynomial. To this end, we first get rid of the denominators

$$
\begin{aligned}
R_2^{\circ}(1+A\tau^2)(1+B\tau^2)(1+C\tau^2)(1+D\tau^2) = {} & (4K1+4K2)\tau(1+A\tau^2)(1+B\tau^2)(1+C\tau^2)(1+D\tau^2) \\
& + K1\tau(1+B\tau^2)(1+C\tau^2)(1+D\tau^2) \\
& + (3K1+3K2)\tau(1+A\tau^2)(1+C\tau^2)(1+D\tau^2) \\
& + 6K1\tau * (1+A\tau^2)(1+B\tau^2)(1+D\tau^2) \\
& + 6K1\tau * (1+A\tau^2)(1+B\tau^2)(1+C\tau^2) \qquad \text{(S2)}
\end{aligned}
$$

and then manipulate the equation to a 9th-order polynomial

$$
\begin{aligned}
&(4K1+4K2)*ABCD{*}\tau^9\\
&-R_2^\circ*ABCD{*}\tau^8\\
&+[(10K1+4K2)ABC+(10K1+4K2)ABD+(7K1+7K2)ACD+(5K1+4K2)BCD]{*}\tau^7\\
&-R_2^\circ(ABC+ABD+ACD+BCD){*}\tau^6\\
&+[(16K1+4K2)AB+(13K1+7K2)AC+(13K1+7K2)AD+(11K1+4K2)BC\\
&+(11K1+4K2)BD+(8K1+7K2)CD]{*}\tau^5\\
&-R_2^\circ(AB+AC+AD+BC+BD+CD){*}\tau^4\\
&+[(19K1+7K2)A+(17K1+4K2)B+(14K1+7K2)C+(14K1+7K2)D]{*}\tau^3\\
&-R_2^\circ(A+B+C+D){*}\tau^2\\
&+(20K1+7K2){*}\tau\\
&-R_2^\circ\\
&=0
\end{aligned}
\tag{S3}
$$

Variations of effective correlation times for the given R_2 value

Deviation from the linear relation between τ_{eff} and R_2 in Eq. 5 in the main text depends on the distribution of timescales, i.e., the weights (α_i) of the timescales (τ_i). To numerically estimate the maximum deviations from the linear behaviour, we searched minima and maxima of Eq. 5 with the fixed τ_{eff} values. We searched extremes using scipy.optimize.minimize package with 200 timescales equally logarithmically spaced between 10^{-20} and 10^{-3} s, and boundary conditions of $\alpha_i \geq 0$ and $\sum_{i=0}^{N} \alpha_i = 1$.

Supplementary results

Contributions of different terms to R_2 values in Eq. 5

The individual terms in Equation 5 are expressed as a function of a single timescale τ as follows

$$y(\tau) = (4K1 + 4K2) * \tau \tag{S4}$$

$$y(\tau) = K1 * \frac{\tau}{1 + A\tau^2} \tag{S5}$$

$$y(\tau) = (3K1 + 3K2) * \frac{\tau}{1 + B\tau^2} \tag{S6}$$

$$y(\tau) = 6K1 * \frac{\tau}{1 + C\tau_i^2} \tag{S7}$$

$$y(\tau) = 6K1 * \frac{\tau}{1 + D\tau^2}, \tag{S8}$$

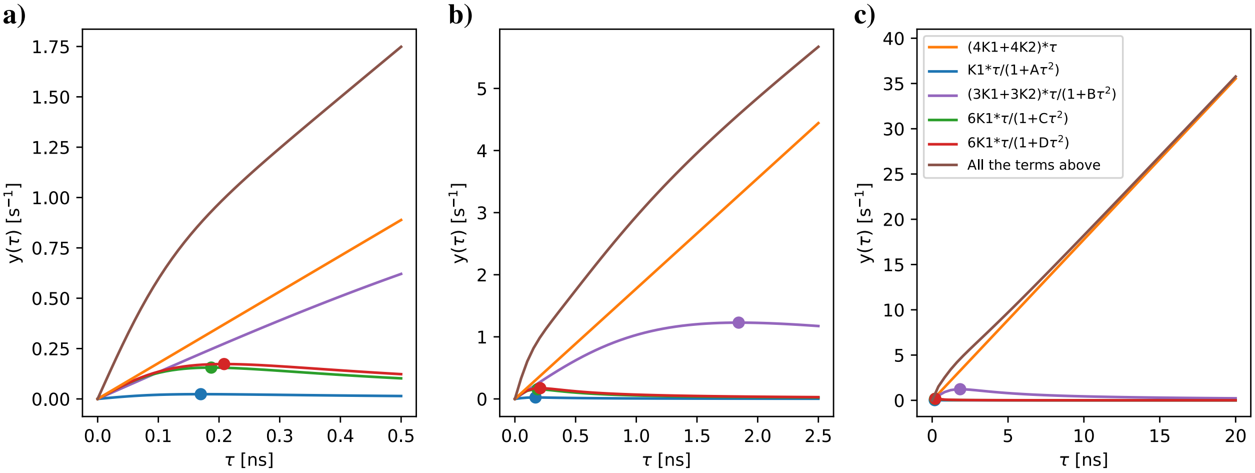

Figure S1: Sizes of linear (orange) and non-linear terms contributing to the R_2 values in Eq. 5 (Eq S4-S8) at 850 MHz with different ranges in axes. a) zoomed to the fastest timescales, b) timescales up to 2.5 ns, c) timescales up to 20 ns. Points show the maxima of individual terms of the Redfield equation.

Accuracy of linear and 1-timescale approximations with respect to MD simulations

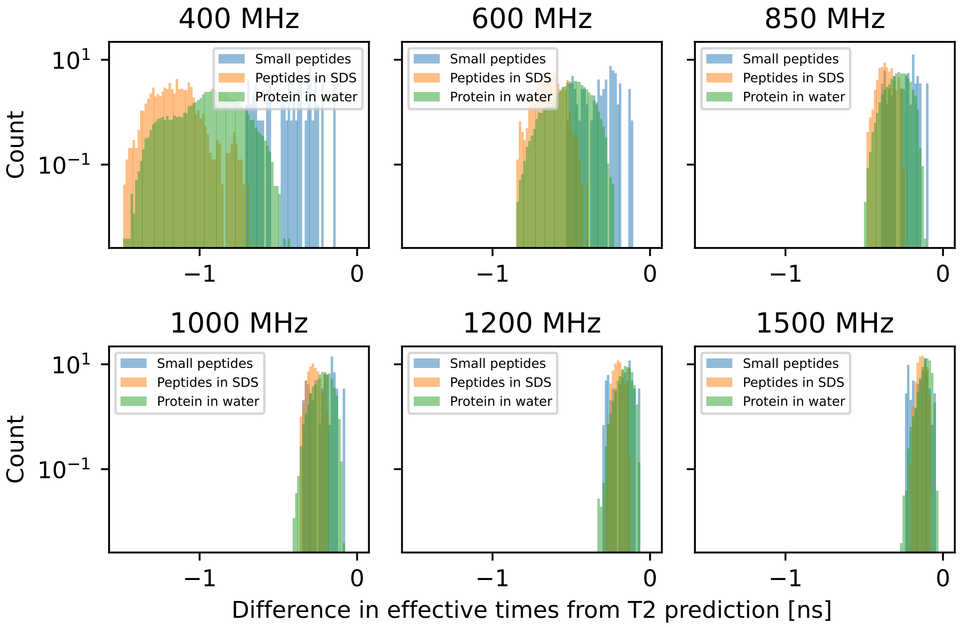

Figure S2: Differences in τ_{eff} values between MD simulations and linear approximation ($\tau_{\text{eff}} = kR_2^0$) at different magnetic fields. The histograms are normalized.

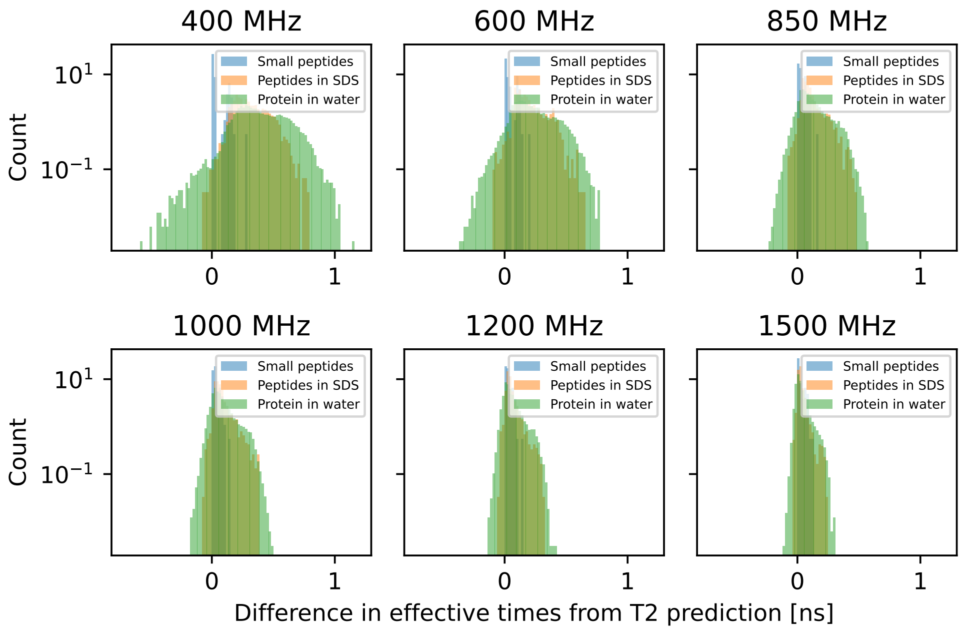

Figure S3: Differences in τ_{eff} values between MD simulations and 1-timescale approximation at different magnetic fields. Histograms are normalized.

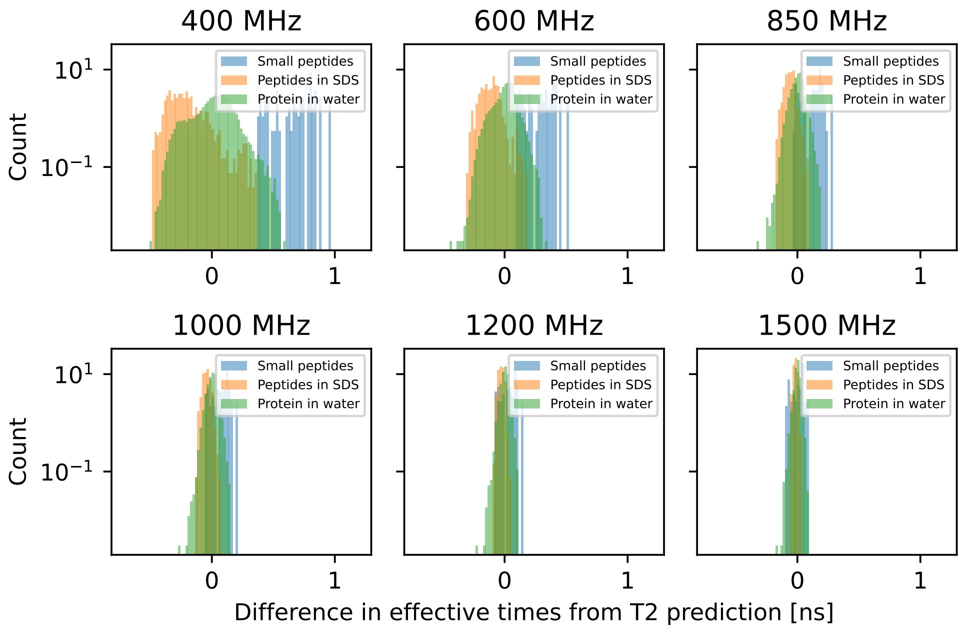

Figure S4: Differences in τ_{eff} values between MD simulations and linear fit ($\tau_{\text{eff}} = m \cdot R_2^0 + c$) at different magnetic fields. The histograms are normalized.

Effective correlation times from linear approximation agree with Lipari-Szabo analysis for globular folded proteins

Rotational motions of folded proteins are typically analyzed using so-called Lipari-Szabo analysis where two rotational timescales are assumed for Eq. 4 in the main text, leading to spectral density equation[9,10]

$$J(\omega) = S^2 \frac{\tau_m}{1+\omega^2\tau_m^2} + (1-S^2)\frac{\tau}{1+\omega^2\tau^2}, \tag{S9}$$

where S is the order parameter measuring spatial restriction of motion, τ_m is the timescale for protein overall motion, and $\tau^{-1} = \tau_{\mathrm{m}}^{-1} + \tau_{\mathrm{i}}^{-1}$ where τ_{i} is the timescale for protein internal motions. An equation approximating timescales of overall motions of folded proteins can be derived from Lipari-Szabo analysis[11]

$$\tau_m = \frac{\sqrt{\frac{6R_2}{R_1} - 7}}{4\pi\omega_{\mathrm{N}}}. \tag{S10}$$

Effective correlation time from this analysis then becomes the weighted average of overall and internal dynamics

$$\tau_{\text{eff}} = S^2 * \tau_m + (1-S^2) * \tau_i. \tag{S11}$$

Figure S5 shows comparison between effective correlation times calculated from linear approximation and Lipari-Szabo approach. For the latter, effective correlation times are calculated using Eq. S11 with typical values for folded proteins, $S^2 \approx 0.9$ and $\tau_i \approx 10\,\mathrm{ps}$, and τ_m calculated from Eq. S10 with R_1 and R_2 values from the literature for folded proteins.[11,12] Values from linear approximation are calculated using the same literature values for R_2. The good agreement between the values indicate that the linear approximation and standard Lipari-Szabo analysis give consistent results for folded proteins.

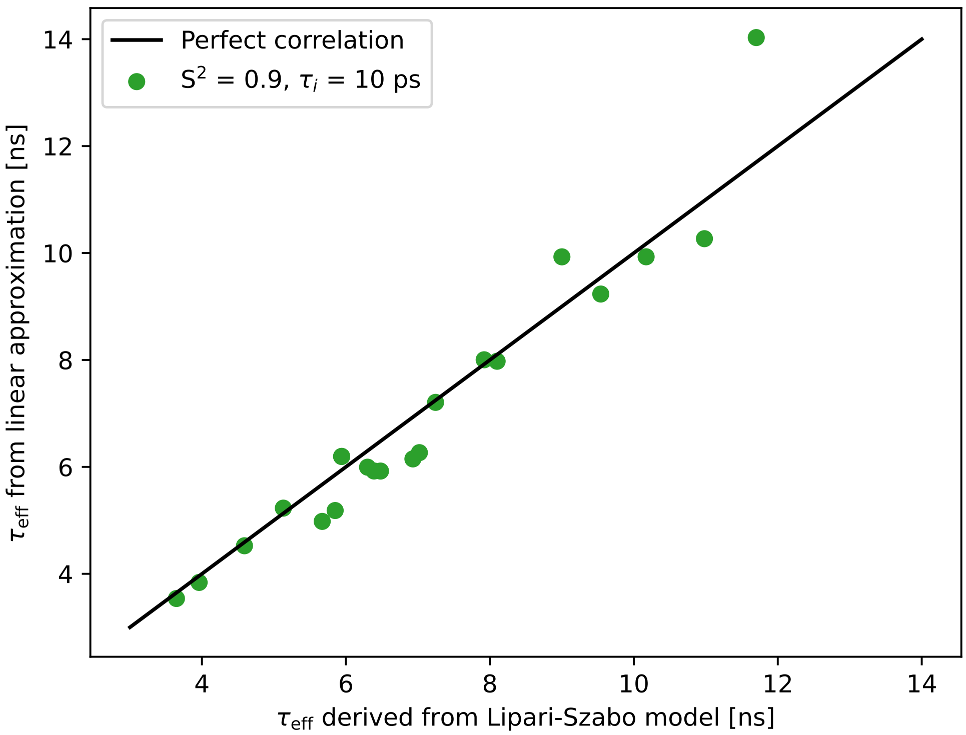

Figure S5: Comparison of the effective correlation times predicted by our linear approximation equation and Lipari-Szabo analysis for the literature data from folded proteins.[11,12] Lipari-Szabo values are calculated using Eqs. S10 and S11.

Magnetic field dependence of R_{ex}

Eq. 6 in the main text provides approximately linear relationship between effective correlation time, τ_{eff}, and R_2^0 when chemical exchange contribution is not present. However, experimental R_2 values substituted to Eq. 6 may contain also a chemical exhange contributions. In this case the equation can be written as

$$\tau_{\mathrm{eff}}^{\mathrm{ex}} = kR_2 = k(R_2^{\circ} + R_{\mathrm{ex}}) = \tau_{\mathrm{eff}} + kR_{\mathrm{ex}}, \tag{S12}$$

where $\tau_{\mathrm{eff}}^{\mathrm{ex}}$ is the resulting effective correlation time when R_2 with chemical exchange contribution is used and τ_{eff} is the effective correlation time when chemical exchange contribution

is assumed to be zero. While τ_{eff} is independent of magnetic field strength, chemical exchange in fast regime makes $\mathrm{R_{ex}}$ dependent on the second power of the magnetic field[13,14] and magnetic field independent exchange factor $\Phi = R_{\mathrm{ex}}/B^2$, is sometimes used.[15]

Assuming that R_{ex} depends on the second power of the magnetic field, relation between R_{ex} and $R_{\mathrm{ex}}^{'}$ in two different magnetic fields B and $B^{'}$, respectively, can be written as

$$R_{\mathrm{ex}}^{'} = \left(\frac{B^{'}}{B}\right)^2 R_{\mathrm{ex}}. \tag{S13}$$

Because τ_{eff} does not depend on magnetic field, Eq. S12 can be written as

$$k(R_2 - R_{\mathrm{ex}}) = k^{'}(R_2^{'} - R_{\mathrm{ex}}^{'}). \tag{S14}$$

Substituting Eq. S13 then gives

$$k(R_2 - R_{\mathrm{ex}}) = k^{'}\left(R_2^{'} - \left(\frac{B^{'}}{B}\right)^2 R_{\mathrm{ex}}\right), \tag{S15}$$

from which R_{ex} can be solved

$$R_{\mathrm{ex}} = \frac{kR_2 - k^{'}R_2^{'}}{k - k^{'}\left(\frac{B^{'}}{B}\right)^2}. \tag{S16}$$

If experimental R_2 and $R_2^{'}$ values are available in two magnetic fields, B and $B^{'}$, field independent τ_{eff} can be then calculated by substituting Eq. S16 into reorganized Eq. S12

$$\tau_{\mathrm{eff}} = k(R_2 - R_{\mathrm{ex}}) = k\left(R_2 - \frac{kR_2 - k^{'}R_2^{'}}{k - k^{'}\left(\frac{B^{'}}{B}\right)^2}\right). \tag{S17}$$

Magnetic field dependence of effective correlation time of B3 domain of protein G

a)
b)
c)

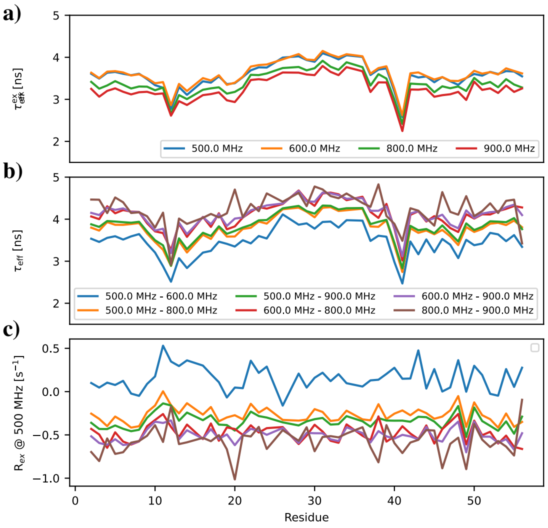

Figure S6: Effective correlation times for B3 domain of protein G determined from R_2 at different fields (BMRB: 26845[16]) (a) without and (b) with the correction term for chemical exchange. (c) The magnitude of chemical exchange term at 500 MHz from our analysis.

References

(1) Nencini, R.; Regnier, M. L. G.; Backlund, S. M.; Mantzari, E.; Dunn, C. D.; Ollila, O. H. S. Probing the dynamic landscape of peptides in molecular assemblies by synergized NMR experiments and MD simulations. *Commun. Chem.* **2024**, *7*, 28.

(2) Sandelin, A.; Nencini, R.; Yasar, E.; Ollila, S. QEBSS: Quality Evaluation Based Simulation Selection for analysis of conformational ensembles and dynamics of multidomain proteins. *ChemRxiv (Physical Chemistry)* **2024**, https://doi.org/doi:10.26434/chemrxiv-2024-h3pmt (accessed 2014-08-17).

(3) Abraham, M. J.; Murtola, T.; Schulz, R.; Páll, S.; Smith, J. C.; Hess, B.; Lindahl, E.

GROMACS: High performance molecular simulations through multi-level parallelism from laptops to supercomputers. *SoftwareX* **2015**, *1-2*, 19–25.

(4) Piana, S.; Robustelli, P.; Tan, D.; Chen, S.; Shaw, D. E. Development of a Force Field for the Simulation of Single-Chain Proteins and Protein-Protein Complexes. *J. Chem. Theory Comput.* **2020**, *16*, 2494–2507.

(5) Piana, S.; Donchev, A. G.; Robustelli, P.; Shaw, D. E. Water dispersion interactions strongly influence simulated structural properties of disordered protein states. *J. Phys. Chem. B* **2015**, *119*, 5113–23.

(6) Mantzari, E.; Sandelin, A. simulations of GGS, GPS and KAPK peptides. 2024; `https://doi.org/10.5281/zenodo.11221462`.

(7) Ollila, O. S.; Heikkinen, H. A.; Iwaï, H. Rotational dynamics of proteins from spin relaxation times and molecular dynamics simulations. *J. Phys. Chem. B* **2018**, *122*, 6559–6569.

(8) Abraham, M.; Alekseenko, A.; Basov, V.; Bergh, C.; Briand, E.; Brown, A.; Doijade, M.; Fiorin, G.; Fleischmann, S.; Gorelov, S. et al. GROMACS 2024.1 Manual. 2024; `https://doi.org/10.5281/zenodo.10721192`.

(9) Lipari, G.; Szabo, A. Model-free approach to the interpretation of nuclear magnetic resonance relaxation in macromolecules. 1. Theory and range of validity. *J. Am. Chem. Soc.* **1982**, *104*, 4546–4559.

(10) Kay, L. E.; Torchia, D. A.; Bax, A. Backbone dynamics of proteins as studied by nitrogen-15 inverse detected heteronuclear NMR spectroscopy: application to staphylococcal nuclease. *Biochemistry* **1989**, *28*, 8972–8979.

(11) Rossi, P.; Swapna, G.; Huang, Y. J.; Aramini, J. M.; Anklin, C.; Conover, K.; Hamil-

ton, K.; Xiao, R.; Acton, T. B.; Ertekin, A. et al. A microscale protein NMR sample screening pipeline. *J. Biomol. NMR* **2010**, *46*, 11–22.

(12) Onwu, S.; Dada, M.; Awojoyogbe, B. Physics and mathematics of magnetic resonance imaging for nanomedicine: An overview. *World J. Transl. Med.* **2014**, *3*, 17–30.

(13) Ishima, R.; Torchia, D. A. Estimating the time scale of chemical exchange of proteins from measurements of transverse relaxation rates in solution. *J. Biomol. NMR* **1999**, *14*, 369–372.

(14) Morin, S. A practical guide to protein dynamics from 15N spin relaxation in solution. *Prog. Nucl. Magn. Reson. Spectrosc.* **2011**, *59*, 245–262.

(15) Ropars, V.; Bouguet-Bonnet, S.; Auguin, D.; Barthe, P.; Canet, D.; Roumestand, C. Unraveling protein dynamics through fast spectral density mapping. *J. Biomol. NMR* **2007**, *37*, 159–177.

(16) Hernández, G.; LeMaster, D. M. Quantifying protein dynamics in the ps–ns time regime by NMR relaxation. *J. Biomol. NMR* **2016**, *66*, 163–174.
