# Supplementary material for: Rapid Interpretation of Protein Backbone Rotation Dynamics Directly from Spin Relaxation Data
Source: J Phys Chem Lett. 2024 Oct 1;15(40):10204–9. doi: 10.1021/acs.jpclett.4c01800 (PMC11480883; doi:10.1021/acs.jpclett.4c01800)
Supplement: Supplementary file 2 — jz4c01800_si_002.pdf [file jz4c01800_si_002.pdf]

jz-2024-01800q.R1

Name: Peer Review Information for "Rapid Interpretation of Protein Backbone Rotation Dynamics Directly from Spin Relaxation Data"

First Round of Reviewer Comments

Reviewer: 1

Comments to the Author

In the present work, <i>Nencini et al</i>. introduce an easy and simplified model to analyze protein dynamics derived from spin relaxation rates. Their work is based on the determination of the residue-wise effective rotational correlation time, exploiting the NMR transverse relaxation rate only. Indeed, the analytical approach rest on the fact that R₂ scales linearly with the effective correlation time (if no conformational exchange is present) and enables the rapid interpretation of protein dynamics. Their approach is applicable to a wide range of structured and unstructured proteins. Moreover, they introduce a correction for conformational exchange that allows the effective correlation time to keep uniform between magnetic fields.

The major advance of the paper is the quick analysis of the transverse relaxation rate and interpretation through the effective correlation time. It does not require any a priori knowledge of the dynamical model and is field independent when a correction for conformational exchange is introduced.

As a consequence, the immediate significance is the possibility to quickly derive residue-wise dynamics with the measurement of NMR transverse relaxation rate at one magnetic field only and may be applicable for a broad scientific community.

Nevertheless, prior to publication, I recommend to the author to work on a major revision:

Although efficient, the current approach suffer from its oversimplification in some cases. For instance the authors observe systematically weaker correlation time in the case of GB3. Care must be taken in such a situation as conformational exchange is not necessarily responsible for this result. Indeed, its has previously been demonstrated that misinterpretation of exchange may introduce bias in the analysis. In GB3, anisotropy of the diffusion tensor, combined with residue-by-

residue variation of the CSA effect clearly affect the results. See for instance Hall et all., JACS, 2006, 128, 7855-7870 and Hall, et al., J. Bomol. NMR., 2003, 27, 261-275. This point has to be discussed prior to publication. The author may check if the introduction of the published residue-by-residue CSA in their analysis (K₂ factor), is prone to solve this issue.

Minor revision:

Page 3: The dipolar coupling constant is badly defined at line 36. Does it read d² or d ?

Page 3: Equation 1-2, the chemical shift anisotropy is defined by ρ in the equations but is defined by Δρ in the text.

Page S3: Eq S2. One must read R<u>2</u>⁰ and not R₂.

Page S4: Boundary conditions to α are negative or equal to zero but the sum of the weights is equal to one. It has to be corrected.

Check carefully the spelling in the entire manuscript, BMRB is written BMBR at several places.

Reviewer: 2

Comments to the Author

This manuscript by Nencini et al. presents an approach to analyze very simply transverse nitrogen-15 relaxation rates in proteins to obtain a single effective correlation time. The amount of work is very significant, combining molecular dynamics simulations and the analysis of many already published experimental datasets. The authors first use MD simulated data to point to transverse relaxation rates as the conventional rate with the easiest relationship to the effective correlation time. They follow by analyzing several published datasets and point to potential inconsistencies between measurements performed at different magnetic fields. One accessible source of inaccuracies originates from the presence of chemical exchange, which is somewhat addressed in the case of the protein CsPinA.

Following editorial recommendations, I will try to address the three following questions:

1. What is the major advance reported in the paper?

The authors present a way to derive a single effective correlation time from the measurement of nitrogen-15 transverse relaxation times. Is this new? One could see this work as a rebranding of existing approaches, that are not mentioned at all in the manuscript.

Indeed the effective correlation times are, by definition, proportional to the spectral density function at zero frequency, J(0). Determining J(0) has been done for three decades following the spectral density mapping approach, which ought to be discussed by the authors. Somehow, there is nothing different between a profile of tau_eff and a profile of J(0). A key advantage of spectral density mapping compared to the approach presented in this manuscript is that, by accounting quantitatively for other spectral density terms, J(0) in spectral density mapping can be determined more faithfully for very mobile systems (peptides, disordered proteins) and rigid domains at the same time.

The authors derive an effective correlation time from transverse relaxation rates, possibly with a simple proportionality relationship. Hence, one could wonder what would be better in a tau_eff profile than a simple R2 profile. The authors mention rightfully in their abstract that the key advantage of their approach is that tau_eff is field independent. This is indeed a true advantage but is the same as a J(0) profile.

The authors add in the abstract that the parameters tau_eff are “intuitively understandable parameters.” I would add that this is a danger, illustrated by the following sentence of the abstract that concludes that the correlation times found in disordered regions support “the view that rotations of disordered and folded regions are uncoupled”. Indeed, in the case of potential weak coupling, tau_eff values would be only marginally altered. I can give an example from the group of Art Palmer, who has analyzed for years the dynamics of the IDR of GCN4 with the extended model-free approach (see Gill et al PCCP 2015 for example). A small order parameter (of the order of 0.1) supports a weak coupling between the motion of the ordered and disordered regions. Whether what is observed is really a coupling of dynamics between the IDR and the folded region or an effect of segmental diffusion of the IDR cannot be addressed by the determination of a single tau_eff value. This important and fundamental question of (un)coupling is much better addressed by the work of the Blackledge group (see Fig 7 in Abyzov et al., JACS 2016) than by the determination of a single tau_eff.

2. What is the immediate significance of this advance?

The immediate significance of this advance comes from Table 1. It is a simple recipe for quickly evaluating the consistency of transverse relaxation rates measured at different magnetic fields or comparing the amount of order between two different proteins/mutants from experiments carried at different magnetic fields. This could be useful but somehow specialized.

If users were to interpret the effective correlation times beyond qualitative comparisons, it is likely that wrong interpretations could be expected.

3. Technical suggestions

I have a series of questions on the choice of transverse relaxation rates. On the one hand, I understand this choice from the fact that this is a routine experiment run for decades in bioNMR laboratories. On the other hand, the choice of transverse relaxation rates comes with the difficulty of the contribution of chemical exchange. Transverse CSA/dipole-dipole cross-correlated cross-relaxation (CCCR) rates are dominated by J(0), like R2 rates, and immune to chemical exchange to a large extent. The methods have been established by the Palmer group in the late 1990's (Kroenke et al. JACS 1998) and improved by Pelupessy (JMR 2003). Work in the past decade by the groups of Ferrage and Blackledge has demonstrated that these could be measured and used quantitatively in IDRs. The TRACT approach even converts an estimate of these rates into a single correlation time (Lee et al. JMR 2006). The latter work should be discussed by the authors and the general use of CSA/DD CCCR rates should be considered.

Parts of the basic theory lack rigor.

In Equations 1 and 2, the authors use "rho" for the CSA and mention "Delta rho" in the text below. I advise the authors to use "Delta sigma" everywhere as is commonly done, especially in solution-state bioNMR.

The definition of d^2_NH is wrong, this is d_NH (not squared). In addition, the third power should be on "r" and not on the subscript "NH".

With respect to r_NH3, the authors write "average cubic length is calculated as ⟨rNH3⟩ = 0.101nm". If ⟨rNH3⟩ is in nm, then a power 3 should be added to ⟨rNH3⟩ in the definition of d_NH.

Also, the commonly used value of r_NH is 102 pm and not 101 pm. This question has been investigated at length by David Case. If I remember well, the debate was whether to use 102 or 104 pm, not 101 pm. Could the authors point to more recent work that makes the use of 101 pm more relevant?

Figure 2 is not very clear to me. What is the difference between black and green points? What are the continuous lines (I cannot understand)? Could the color code for different proteins be more explicit?

Figure 2 also illustrates issues with the contribution of chemical exchange to transverse relaxation rates. A solution would be the use of CCCR rates (see above). The authors have chosen to use pairwise comparisons of transverse relaxation rates. This is somehow a strange choice as the authors use a dataset with measurements at 4 different fields. To me, Figure 4a clearly points at inconsistencies between the measurement at 700 MHz and at lower fields. Maybe this was done at another temperature or on a sample with a different concentration. The data at other fields should be used together to maximize the amount of information and reduce the very large noise exposed in Figure 4b. The very low and artefactual values from the comparisons of rates at 600 and 700 MHz show the limitations of this approach. Also, the mostly negative values of Rex from the comparison of rates at 400 and 500 MHz is not physical. In a fitting procedure the authors should rightfully exclude negative values of Rex and address the question of the quality of the fit in a second time.

The reference to silk proteins and materials is somehow confusing. Are the authors suggesting that their approach can be used in solid-state NMR? Did I misunderstand this statement?

On page S4 of the supporting information, why do the authors choose a range of correlation times so broad? I imagine that 1 ps to 1 us would be already comfortably large.

In Equation S11, the authors use the order parameter, should they use the squared order parameter S_2?

In figure S5, the choice of S2 = 0.9 and tau_i = 10 ps points to a very rigid NH pair. I have to admit I do not really understand what is shown there. Which R2 are used? What values of tau_c are used?

In a similar line, a calculation with the same overall tau_c and varying S2 and tau_i would be very interesting.

Author's Response to Peer Review Comments:

Dear editor,

we thank you for considering our manuscript for publication in The Journal of Physical Chemistry Letters. Please find detailed reply to reviewers' comments enclosed. We have also implemented all non-scientific modifications that are mentioned in the Manuscript Formatting Request. The changes to the main text and supplementary information are marked in enclosed latexdiff.pdf and latexdiffSI.pdf files.

Reviewer: 1
In the present work, Nencini et al. introduce an easy and simplified model to analyze protein dynamics derived from spin relaxation rates. Their work is based on the determination of the residue-wise effective rotational correlation time, exploiting the NMR transverse relaxation rate only. Indeed, the analytical approach rest on the fact that R₂ scales linearly with the effective correlation time (if no conformational exchange is present) and enables the rapid interpretation of protein dynamics. Their approach is applicable to a wide range of structured and unstructured proteins. Moreover, they introduce a correction for conformational exchange that allows the effective correlation time to keep uniform between magnetic fields.

The major advance of the paper is the quick analysis of the transverse relaxation rate and interpretation through the effective correlation time. It does not require any a priori knowledge of the dynamical model and is field independent when a correction for conformational exchange is introduced.

As a consequence, the immediate significance is the possibility to quickly derive residue-wise dynamics with the measurement of NMR transverse relaxation rate at one magnetic field only and may be applicable for a broad scientific community.

REPLY:
We thank reviewer for positive comments.

Reviewer: 1
Nevertheless, prior to publication, I recommend to the author to work on a major revision:

Although efficient, the current approach suffer from its oversimplification in some cases. For instance the authors observe systematically weaker correlation time in the case of GB3. Care must be taken in such a situation as conformational exchange is not necessarily responsible for this result. Indeed, its has previously been demonstrated that misinterpretation of exchange may introduce bias in the analysis. In GB3, anisotropy of the diffusion tensor, combined with residue-by-residue variation of the CSA effect clearly affect the results. See for instance Hall et all., JACS, 2006, 128, 7855-7870 and Hall, et al., J. Bomol. NMR., 2003, 27, 261-275. This point has to be discussed prior to publication. The author may check if the introduction of the published residue-by-residue CSA in their analysis (K₂ factor), is prone to solve this issue.

REPLY:
It is not fully clear to what reviewer is referring with this “*...authors observe systematically weaker correlation time in the case of GB3*”. Nevertheless, we assume that the reviewer refers to this sentence in the manuscript: “*for the B3 domain of protein G, we observed systematically slightly lower* τ_{eff} *values with 800 MHz than with other fields that cannot be explained by the chemical exchange correction*

(supplementary Fig. S6)." When replying to this comment, we realized that the 900 MHz data was incorrectly shown in top panel of Fig. S6. After correcting this, we have updated the sentence in the manuscript to "*...for the B3 domain of protein G, we observed systematically slightly lower τ_{eff} values with 800 MHz and 900 MHz than with 500 MHz and 600 MHz that cannot be explained by the chemical exchange correction (supplementary Fig. S6)*". Reviewer is presumably suggesting that this could arise from anisotropy of the diffusion tensor or residue-by-residue variations in CSA.

In Figure 1 in the manuscript, we demonstrate the linear relation between R_2 and effective correlation times for wide range of proteins from peptides in micelles to partially disordered proteins. We have now emphasized the heterogeneity of proteins used in Fig. 1 by adding visualizations of representative conformations into the figure. Many of these proteins do not even have well defined diffusion tensors due to significant disorder. Therefore, it seems unlikely that anisotropic diffusion tensor would cause the lower effective correlation times at 800 MHz and 900 MHz. Furthermore, it would be difficult to explain why this effect would be magnetic field dependent.

For CSA, the average value (-160 ppm in Eqs. 1-3) is used in the analysis for all residues. Residue-byresidue variation of CSA should not systematically shift the results but rather cause differences between residues. Because differences between fields in Fig. S6 are systematic, it seems unlikely that they would arise from residue-by-residue variations in CSA. Nevertheless, we estimated the effect of residue specific CSA by substituting experimental residues specific values to K2 in Eq. (5) and recalculated the results shown in Fig. S6 a). We used the CSA values from Yao et al. (https://pubs.acs.org/doi/10.1021/ja910186u), which are more recent and have smaller errors than in the articles mentioned by the reviewer. Figure 1 below shows some variation between residues, but does not explain the differences observed between magnetic fields. Therefore, we do not find any evidence that residue-by-residue variations in CSA would affect to our analysis.

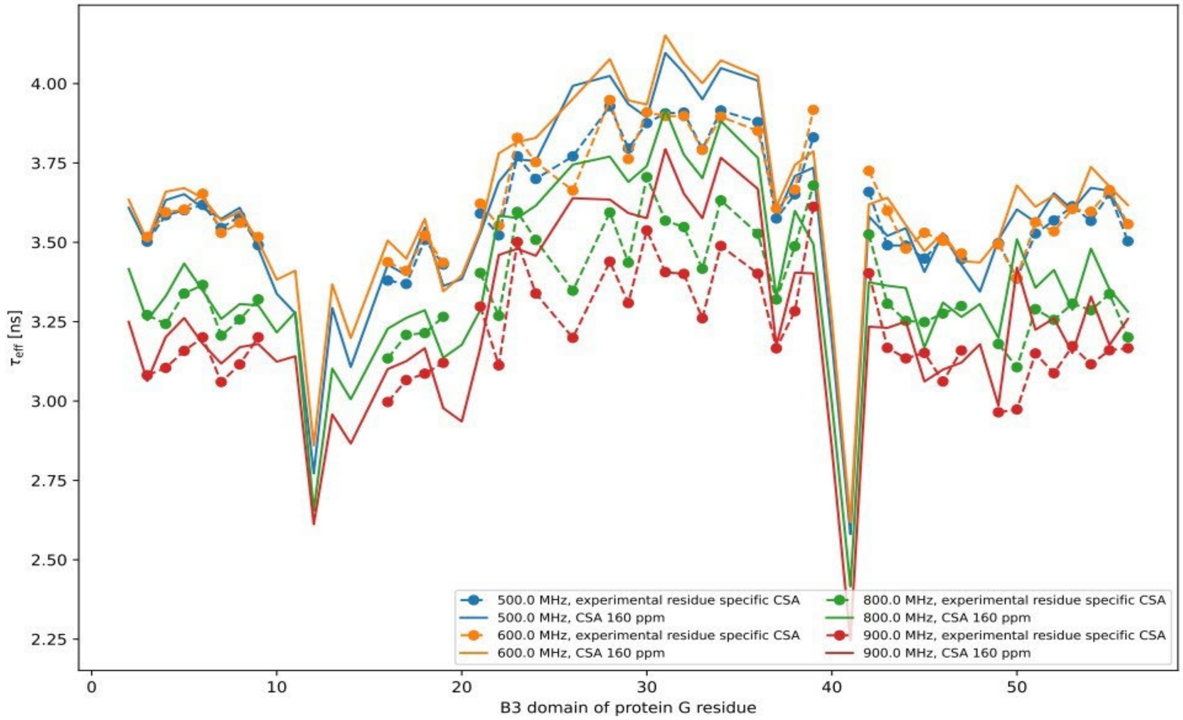

Figure 1: Effect of residue-by-residue variations of CSA (Yao et al. https://pubs.acs.org/doi/10.1021/ja910186u) to effective correlations times of GB3 measured in different magnetic field strengths.

Reviewer: 1
Minor revision:

Page 3: The dipolar coupling constant is badly defined at line 36. Does it read d² or d ?

REPLY:
We thank reviewer for pointing out this and other typos in the manuscript. This is now corrected.

Reviewer: 1
Page 3: Equation 1-2, the chemical shift anisotropy is defined by ρ in the equations but is defined by Δρ in the text.

REPLY:
We have now changed this to “Delta sigma” everywhere as suggested by the Reviewer 2.

Reviewer: 1
Page S3: Eq S2. One must read R<u>2</u>⁰ and not R₂.

REPLY:
This is now corrected in Eq. S2 and also in Eq. S3.

Reviewer: 1
Page S4: Boundary conditions to α are negative or equal to zero but the sum of the weights is equal to one. It has to be corrected.

REPLY:
Boundary conditions to α should be positive or equal to zero. This typo is now corrected.

Reviewer: 1
Check carefully the spelling in the entire manuscript, BMRB is written BMBR at several places.

REPLY:
These are now corrected.

Reviewer: 2
This manuscript by Nencini et al. presents an approach to analyze very simply transverse nitrogen-15 relaxation rates in proteins to obtain a single effective correlation time. The amount of work is very significant, combining molecular dynamics simulations and the analysis of many already published experimental datasets. The authors first use MD simulated data to point to transverse relaxation rates as the conventional rate with the easiest relationship to the effective correlation time. They follow by analyzing several published datasets and point to potential inconsistencies between measurements performed at different magnetic fields. One accessible source of inaccuracies originates from the presence of chemical exchange, which is somewhat addressed in the case of the protein CsPinA.

REPLY:

We thank reviewer for constructive comments.

Reviewer: 2
Following editorial recommendations, I will try to address the three following questions:

1. What is the major advance reported in the paper?

The authors present a way to derive a single effective correlation time from the measurement of nitrogen-15 transverse relaxation times. Is this new? One could see this work as a rebranding of existing approaches, that are not mentioned at all in the manuscript.

Indeed the effective correlation times are, by definition, proportional to the spectral density function at zero frequency, J(0). Determining J(0) has been done for three decades following the spectral density mapping approach, which ought to be discussed by the authors. Somehow, there is nothing different between a profile of tau_eff and a profile of J(0).

REPLY:
We agree with the reviewer that J(0) could be determined from the spectral density mapping and then linked to the effective correlation time. We believe that such rebranding of spectral density mapping would make it more comprehensible for broader audience and would be therefore highly valuable. However, this is not what we do here.

Here we numerically demonstrate for wide range of systems that the effective correlation time can be determined directly from R_2 values without spectral density mapping or other further analyses. Advantage of our approach is the smaller number of required experimental parameters to derive the effective correlation time than in the spectral density mapping. In spectral density mapping, J(0) is solved from a set of equations that require 6 or 3 (reduced spectral density mapping) experimental parameters for each residue. In our approach, the effective correlation time is solved only from one experimental parameter per residue using the linear equation. This requires less experimental data and is numerically more simple than the spectral density mapping. We have now clarified this by adding a sentence into the second last paragraph of the manuscript: "*Similar analyses might be possible by determining J(0) values from spectral density mapping,[38,39] but this would require 3-6 experimental parameters per residue while our approach requires only R_2 values.*"

Reviewer: 2

A key advantage of spectral density mapping compared to the approach presented in this manuscript is that, by accounting quantitatively for other spectral density terms, J(0) in spectral density mapping can be determined more faithfully for very mobile systems (peptides, disordered proteins) and rigid domains at the same time.

REPLY:
In Figure 1 in the manuscript we demonstrate that our approach is valid for wide range of proteins including peptides in micelles and partially disordered proteins. We have now emphasized this by adding visualizations of representative conformations of used proteins into Figure 1. Furthermore, from our unpublished data we see that our approach works also for peptide stabilized nanodiscs. Therefore our approach seems to work for wide range of systems and advantage of spectral density mapping would be quite limited in this respect.

Reviewer: 2
The authors derive an effective correlation time from transverse relaxation rates, possibly with a simple proportionality relationship. Hence, one could wonder what would be better in a tau_eff profile than a simple R2 profile. The authors mention rightfully in their abstract that the key advantage of their approach is that tau_eff is field independent. This is indeed a true advantage but is the same as a

J(0) profile.

REPLY:
Physical meanings of J(0) or R_2 are clear only for very advanced experts in detecting biomolecular dynamics with NMR. In contrast, effective correlation times provide average timescales for rotational motions of protein backbones. These are intuitively understandable parameters for significantly wider audience than J(0) or R_2. Therefore, we believe that the main advantage of effective correlation time over J(0) is its comprehensibility beyond NMR experts.

Reviewer: 2
The authors add in the abstract that the parameters tau_eff are "intuitively understandable parameters." I would add that this is a danger, illustrated by the following sentence of the abstract that concludes that the correlation times found in disordered regions support "the view that rotations of disordered and folded regions are uncoupled". Indeed, in the case of potential weak coupling, tau_eff values would be only marginally altered. I can give an example from the group of Art Palmer, who has analyzed for years the dynamics of the IDR of GCN4 with the extended model-free approach (see Gill et al PCCP 2015 for example). A small order parameter (of the order of 0.1) supports a weak coupling between the motion of the ordered and disordered regions. Whether what is observed is really a coupling of dynamics between the IDR and the folded region or an effect of segmental diffusion of the IDR cannot be addressed by the determination of a single tau_eff value. This important and fundamental question of (un)coupling is much better addressed by the work of the Blackledge group (see Fig 7 in Abyzov et al., JACS 2016) than by the determination of a single tau_eff.

REPLY:
We agree with the reviewer that uncoupling of rotational motions cannot be addressed by single effective correlation time values. Instead of using single effective correlation times, we compare here dynamics between partially disordered proteins that have folded and unfolded domains with different lengths.

If rotations of folded and unfolded regions were coupled, dynamics of unfolded regions would slow down upon increase of folded domain size. This is not observed in our manuscript as stated in page 8 "*Fig. 2 shows that the total effective correlation times of folded regions vary between approximately 5 ns and 17 ns with a slight dependence on the size of the region (Pearson correlation coeffient 0.66, p-value 0.05). On the other hand, effective correlation times of disordered regions are approximately 1-2 ns, independently of the size of the protein or the disordered part (Pearson correlation coeffient 0.17, p-value 0.66)*". This is suggesting that dynamics of unfolded regions are uncoupled from folded regions in the studied proteins.

We have now further emphasized these results by adding the Pearson correlation coefficients and lines into Figure 2. The correlation coefficients are now mentioned also in the text.

As pointed out by the reviewer alternative strategy would be to make experiments with different lengths of disordered proteins attached to folded region. In addition to article by Abyzov et al. mentioned by the reviewer, some of us have done this in our previous studies for TonB protein (Refs. 3 and 27) where only weak coupling between folded and unfolded regions was observed. The approach presented in our manuscript enables comparisons of protein dynamics between proteins with different sizes using readily available experimental data in different fields. This is substantially easier than performing new experiments with different lengths of disordered regions attached to folded regions.

Therefore, we still believe that our results demonstrate that the dynamics of folded and unfolded regions are uncoupled in the proteins studied here, similarly to TonB. However, as pointed out by the reviewer, caution should be exercised when extrapolating this result to other proteins. Therefore, we have slightly specified our conclusions related to this.

Last sentence of the abstract
"*… supporting the view that rotations of disordered and folded regions are uncoupled.*" is updated to
"*… suggesting that rotations of disordered and folded regions are uncoupled in these proteins.*" and
Sentence in page 8
"*This suggests a conception for partially disordered proteins where dynamics of folded and disordered parts are generally uncoupled. This is in line with previous results that draw similar conclusions for TonB protein using NMR experiments and MD simulations.*[3,27]" is updated to
"*This suggests that dynamics of folded and disordered parts are uncoupled in these proteins similarly to*
TonB protein that is previously analyzed by combining NMR experiments and MD simulations.[3,27]"

Reviewer: 2
2. What is the immediate significance of this advance?

The immediate significance of this advance comes from Table 1. It is a simple recipe for quickly evaluating the consistency of transverse relaxation rates measured at different magnetic fields or comparing the amount of order between two different proteins/mutants from experiments carried at different magnetic fields. This could be useful but somehow specialized.

REPLY:
In addition to the immediate significant advances mentioned by the reviewer, our results also enable rapid estimation of intuitively understandable effective correlation time values directly from R_2 values. This enables rapid interpretation of protein backbone dynamics not only for NMR experts, but also for scientists who are not very familiar with spin relaxation experiments. We believe that this is a significant immediate advance with broad interest.

Reviewer: 2
If users were to interpret the effective correlation times beyond qualitative comparisons, it is likely that wrong interpretations could be expected.

REPLY:

It is not clear which kind of wrong interpretations the reviewer is expecting here. As any parameter, it is possible to misinterpret effective correlation times. However, we refrain from further speculation here as we are not sure what the reviewer is expecting here.

Reviewer: 2
3. Technical suggestions

I have a series of questions on the choice of transverse relaxation rates. On the one hand, I understand this choice from the fact that this is a routine experiment run for decades in bioNMR laboratories. On the other hand, the choice of transverse relaxation rates comes with the difficulty of the contribution of chemical exchange. Transverse CSA/dipole-dipole cross-correlated cross-relaxation (CCCR) rates are dominated by J(0), like R2 rates, and immune to chemical exchange to a large extent. The methods have been established by the Palmer group in the late 1990's (Kroenke et al. JACS 1998) and improved by Pelupessy (JMR 2003). Work in the past decade by the groups of Ferrage and Blackledge has demonstrated that these could be measured and used quantitatively in IDRs. The TRACT approach even converts an estimate of these rates into a single correlation time (Lee et al. JMR 2006). The latter work should be discussed by the authors and the general use of CSA/DD CCCR rates should be considered.

REPLY:
As pointed out by the reviewer, the transverse relaxation parameters, R_2, are measured routinely by many groups performing protein NMR experiments. BMRB database (https://bmrb.io/) contains R_2 values for 337 proteins. On the other hand, CCCR values are measured much less often and they are not even listed as parameters available in the BMRB database. For proteins shown in Fig. 2, CCCR values are reported only for EN2 and BRD4 proteins. Therefore, using R_2 values instead of CCCR provides significantly wider scope for the study. This applies to the data in this manuscript (7 systems analyzed in Fig. 2 instead of 2) as well as to future studies (e.g. analysis can be performed for all proteins in BMRB with R_2 values available).

In the manuscript we discuss two cases (CsPinA in Fig. 4 and GB3 in Fig. S6) where chemical exchange potentially has some influence on the results. We were not able to find literature CCCR data for neither of these proteins. Therefore, even if similar approach would work for CCCR data and it would be free from chemical exchange effect, it could not be used for these proteins without further experiments.

Lee et al. Indeed convert TRACT experiments to a single correlation time. However, for this they assume isotropic rigid body rotation for a protein. Therefore, their correlation time is different to the effective correlation time presented in our manuscript, as they also recognize by stating "*The value thus obtained for τ_c represents a lower limit for the overall rotational correlation time, due to the rigid body assumption used*" after Eq. (6) in their article. Validity of this assumption would be questionable especially for disordered protein regions.

In order to use CCCR in similar fashion as R_2, similar numerical studies should be performed to ensure that it also has a linear relation with effective correlation time. This would be interesting and probably successful. However, as discussed in this reply, we do not expect major immediate advantages from this analysis in comparison with the current manuscript. Therefore, we have left this for further studies.

Reviewer: 2

Parts of the basic theory lack rigor.
In Equations 1 and 2, the authors use "rho" for the CSA and mention "Delta rho" in the text below. I advise the authors to use "Delta sigma" everywhere as is commonly done, especially in solution-state bioNMR.

REPLY:
We thank reviewer for pointing out this, as well as other typos and relevant details in the manuscript. We now use "Delta sigma" as suggested by the reviewer.

Reviewer: 2
The definition of d^2_NH is wrong, this is d_NH (not squared). In addition, the third power should be on "r" and not on the subscript "NH".

REPLY:
These are now corrected.

Reviewer: 2
With respect to r_NH3, the authors write "average cubic length is calculated as rNH3 = 0.101nm". If⟨ ⟩ ⟨rNH3 is in nm, then a power 3 should be added to rNH3⟩ ⟨ ⟩ in the definition of d_NH.
Also, the commonly used value of r_NH is 102 pm and not 101 pm. This question has been investigated at length by David Case. If I remember well, the debate was whether to use 102 or 104 pm, not 101 pm.
Could the authors point to more recent work that makes the use of 101 pm more relevant?

REPLY:
After checking the literature based on the reviewer's comments, we realized that it would be indeed better to use slightly larger values values (e.g. from here https://doi.org/10.1021%2Fja805654f). However, the difference is in third significant digit and should not therefore affect the conclusions of this work. We thank reviewer for pointing this out and we will correct this in our code for future use. However, we will not reanalyze the results in this manuscript because significant effect conclusions is not expected due to small differences between the used and proposed values.

Reviewer: 2
Figure 2 is not very clear to me. What is the difference between black and green points? What are the continuous lines (I cannot understand)? Could the color code for different proteins be more explicit?

REPLY:
Missing details are now clarified in the caption of Figure 2.

Reviewer: 2
Figure 2 also illustrates issues with the contribution of chemical exchange to transverse relaxation rates. A solution would be the use of CCCR rates (see above). The authors have chosen to use pairwise comparisons of transverse relaxation rates. This is somehow a strange choice as the authors use a dataset with measurements at 4 different fields. To me, Figure 4a clearly points at inconsistencies between the measurement at 700 MHz and at lower fields. Maybe this was done at another temperature or on a sample with a different concentration. The data at other fields should

be used together to maximize the amount of information and reduce the very large noise exposed in Figure 4b. The very low and artefactual values from the comparisons of rates at 600 and 700 MHz show the limitations of this approach. Also, the mostly negative values of Rex from the comparison of rates at 400 and 500 MHz is not physical. In a fitting procedure the authors should rightfully exclude negative values of Rex and address the question of the quality of the fit in a second time.

REPLY:
We assume that the reviewer is referring to Figure 4 (instead of figure 2) everywhere in this comment. When replying to this comment, we indeed found a numerical mistake from our plotting script for 700 MHz data.
This is now corrected and systematic inconsistency for this field is not present in the figure anymore.

However, the magnetic field dependence for residues around 50, that are explained by the chemical exchange contribution in the manuscript, still remain.

Reviewer is suggesting to use CCCR rates to resolve this. However, we have not found CCCR values for this protein from the literature, thus they would not be a solution in this case without further experiments.

Reviewer is wondering why pairwise comparison is done although values are from 4 different fields. We have derived Eq. S17 in the SI for the pairwise comparison. We are not aware of any other ways to do this comparison pairwise or simultaneously using multiple fields.

There are indeed some artificially low values in Fig. 4 b and unphysical negative values betweeen 400500MHz. However, we believe that these arise from fluctuations in the original experimental data rather than limitations of this approach. Notably, such fluctuations are not observed in figure S6 showing the similar data for other protein, supporting the argument that these fluctuation arise from original data rather than the used approach. R_{ex} values are not used in any fitting procedure, so we are not sure where reviewer is referring the with last sentence in this comment.

Reviewer: 2
The reference to silk proteins and materials is somehow confusing. Are the authors suggesting that their approach can be used in solid-state NMR? Did I misunderstand this statement?

REPLY:
Silk proteins are partially disordered proteins that are soluble also in monomeric form, and can be therefore studied also with solution state NMR. To clarify this, we have changed the reference related to silk proteins from a general review to an article where silk proteins have been studied using solution state NMR spin relaxation experiments (https://doi.org/10.1038/s41467-018-04570-5). One of use has previously published an article regarding the determination of effective correlation times from solid state experiments (http://dx.doi.org/10.1063/1.4906274), but the approach in the current article is not applicable to solid state
NMR.

Reviewer: 2
On page S4 of the supporting information, why do the authors choose a range of correlation times so broad? I imagine that 1 ps to 1 us would be already comfortably large.

REPLY:

Macromolecules can exhibit dynamics with hundreds of microsecond timescales (for example exchange between two conformations). On the other hand, dynamics faster than picosecond are present in biomolecular systems. Therefore, we have decided to use rather too large than small range here.

Reviewer: 2
In Equation S11, the authors use the order parameter, should they use the squared order parameter S_2?

REPLY:
This is now corrected.

Reviewer: 2
In figure S5, the choice of S2 = 0.9 and tau_i = 10 ps points to a very rigid NH pair. I have to admit I do not really understand what is shown there. Which R2 are used? What values of tau_c are used?

REPLY:
Key message of the this figure is stated in Page 8 in the manuscript: "*For folded proteins, results from the linear approximation presented in this work are in line with the standard Lipari-Szabo analysis*[28] *in supplementary figure S5*". We show this data to demonstrate that our results are in line with the widely used Lipari-Szabo approach for folded proteins. Because this concerns folded proteins, we use here typical values for folded proteins which indeed have relatively rigid N-H bonds.

We have now clarified this by changing the title of the corresponding section in SI to "*Effective correlation times from linear approximation agree with Lipari-Szabo analysis for globular folded proteins*". Furthermore, this section and figure S5 caption in the SI are now substantially clarified.

Reviewer: 2
In a similar line, a calculation with the same overall tau_c and varying S2 and tau_i would be very interesting.

REPLY:
Figure S5 is demonstrating the consistency of analyzing experimental data for folded proteins with these two approaches. Setting directly all values in Eq. S11 without any experimental values would give trivially the same results.

jz-2024-01800q.R2

Name: Peer Review Information for "Rapid Interpretation of Protein Backbone Rotation Dynamics Directly from Spin Relaxation Data"

Second Round of Reviewer Comments

Reviewer: 1

Comments to the Author

The authors have addressed all my concerns. Nevertheless, they should mention they have tried to introduce the CSA effect in the case of GB3 and show their plot in supplementary materials.

Author's Response to Peer Review Comments:

Dear Professor Christy Landes,

thank you for considering our manuscript for The Journal of Physical Chemistry Letters.

We have now made all the requested non-scientific changes.

Regarding licenses for graphics, the illustration of NMR instrument in the TOC figure is created by Biorender. We have now added the license information in the Acknowledgements section. The license is also included in the "Supporting Information for Review Only." files. Other copyrighted figures are not included in the manuscript.

BR,

Samuli Ollila
